# Cyclosporine A: a valid candidate to treat COVID-19 patients with acute respiratory failure?

**DOI:** 10.1186/s13054-020-03014-1

**Published:** 2020-06-02

**Authors:** Martin Cour, Michel Ovize, Laurent Argaud

**Affiliations:** 1 Hospices Civils de Lyon, Service de Médecine Intensive-Réanimation, Hôpital Edouard Herriot, 5, place d’Arsonval, 69437 Lyon Cedex 03, France; 2 INSERM UMR1060 (CarMeN), Université de Lyon, Lyon, France; 3 Hospices Civils de Lyon, Centre d’Investigation Clinique de Lyon, Université de Lyon, Lyon, France

Coronavirus disease 2019 (COVID-19) caused by severe acute respiratory syndrome coronavirus 2 (SARS-CoV-2) has led to an unprecedented number of hypoxemic pneumoniae since the first cases were diagnosed in China in December 2019. In only a few weeks, tens of thousands of patients have died of acute respiratory failure (ARF) in Europe and then in North America, according to Johns Hopkins University and Medicine Coronavirus Resource Center (https://coronavirus.jhu.edu).

Numerous promising antiviral therapies against SARS-CoV-2 are being investigated with the hope of preventing both interindividual transmission and severe complications of the disease [1]. COVID-19-induced ARF is thought to be related to both direct viral pathogenicity and dysregulated inflammatory host response. Unfortunately, current treatment remains only supportive and symptomatic [2]. Therefore, there is an urgent need for effective drugs targeting this life-threatening complication, in particular, for patients developing acute respiratory distress syndrome. The best candidate drug should (1) prevent hyperinflammation-induced lung injury; (2) inactivate viral replication; (3) be widely available; (4) be safe, including when administered with other antivirals; and (5) be affordable. We speculate that cyclosporine A (CsA) might fulfill all these criteria.

CsA has been used for decades to prevent organ rejection and to treat T cell-associated autoimmune diseases such as rheumatoid arthritis, systemic lupus erythematosus, or interstitial lung disease [3, 4]. CsA exerts its immunosuppressive and anti-inflammatory effects by binding to cyclophilin-A (Cyp-A) which prevents the nuclear factor of activated T cell (NF-AT) activation and the transcription of genes required for T cell proliferation, notably interleukin-2 (Fig. 1) [3, 4]. Interestingly, SARS-CoV non-structural protein 1 was found to induce the expression of interleukin-2 via NF-AT activation [5, 6], which might trigger the cytokine storm seen in patients with severe COVID-19 [1]. Consequently, it is tempting to use CsA to dampen the dysregulated immune response in the setting of COVID-19-related ARF. In addition, a major advantage of CsA over most anti-inflammatory drugs lies in its potent antiviral activity against coronaviruses (Fig. 1). Indeed, at low micromolar and non-cytotoxic concentrations, CsA blocks the replication of all coronavirus genera (including SARS-CoV-1) in cell cultures [5, 6]. This antiviral property is thought to be mediated by the inhibition of Cyp-A-dependant viral assembly as well as inhibition of the NF-AT pathway [5, 6]. Finally, equally important, CsA binds to Cyp-D, which inhibits opening of the mitochondrial permeability transition pore (mPTP), a pathophysiological event triggered by injury (e.g., oxidative stress, hypoxia, and ischemia/reperfusion) that may compromise cell function or survival (Fig. 1) [3]. In addition to preventing cell death under stress conditions [3], genetic or pharmacological specific inhibition of Cyp-D has the potential to hinder viral replication [6].

**Fig. 1:**
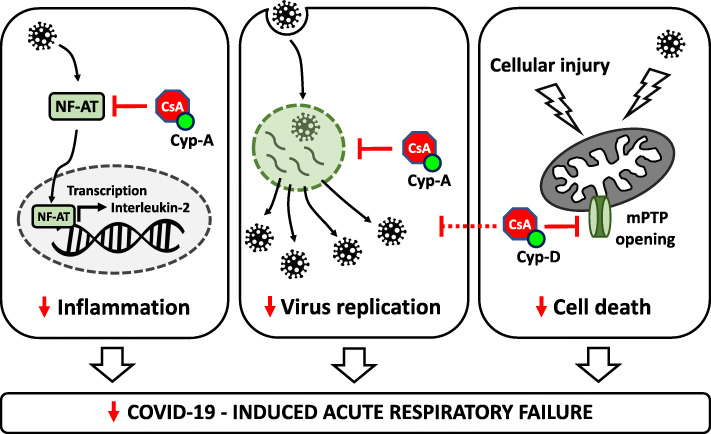
Schematic overview of the presumed protective effects of cyclosporine A in COVID-19-induced acute respiratory failure. Cyclosporine A (CsA), binding to cyclophilin A (Cyp-A), prevents the translocation of nuclear factor of activated T cells (NF-AT) into the nucleus (left box) and blocks viral replication (middle box) and thus transcription of pro-inflammatory cytokines (e.g., interleukin-2). CsA, binding to cyclophilin D (Cyp-D), also prevents mitochondrial permeability transition pore (mPTP) opening-induced injury and thus cell death/dysfunction (right box). The red color is used to indicate the effects of CsA

In experimental models of sepsis and/or inflammation-induced acute lung injury, CsA has been consistently reported to improve lung function via mitochondrial processes, including PTP inhibition [7, 8]. Even though no clinical trial has been specifically designed to investigate the potential benefits of CsA in ARF, we reported, in a post hoc analysis of the *CsA in cardiac arrest resuscitation* (CYRUS) trial, that CsA may dramatically limit the severity of post-cardiac arrest ARF, corroborating the abovementioned pre-clinical findings [9, 10]. Encouragingly, we also observed, in a predefined ancillary study of the CYRUS trial, significantly higher total and CD4+ lymphocyte counts at 24 h after cardiac arrest in patients treated with CsA than in controls [11]. Importantly, no safety concerns, including an increase in nosocomial infections, were reported in trials (in which thousands of patients were included) that have tested short-term off-label CsA use, as it would be the case for COVID-19 [3, 9–13]. Yet, the toxicity of CsA cannot be excluded at concentrations that may be required to inhibit SARS-CoV-2 [3–6]. This potential issue could be overcome using inhaled CsA, providing high lung tissue exposure (with minimal increase in plasma concentration), as it has been done safely and effectively after lung transplantation [14, 15]. Eventually, CsA is not expensive and might be used worldwide, including in countries where the COVID-19 health crisis is rapidly growing with little or no access to expensive therapies. Moreover, none of the antivirals against SARS-CoV-2 currently under investigation is contraindicated in combination with CsA.

To summarize, CsA has the potential to prevent (1) uncontrolled inflammatory response, (2) SARS-CoV-2 replication, and (3) acute lung injury. We believe that there is a solid rationale for investigating whether CsA might bring clinical benefit in COVID-19 patients with ARF.

## Data Availability

Not applicable.

## Abbreviations

ARF: Acute respiratory failure
COVID-19: Coronavirus disease 2019
CsA: Cyclosporine A
Cyp: Cyclophilin
CYRUS: *Cyclosporine A in cardiac arrest resuscitation trial*
mPTP: Mitochondrial permeability transition pore
NF-AT: Nuclear factor of activated T cells
SARS-CoV: Severe acute respiratory syndrome coronavirus
